# Neuropeptide stimulation of physiological and immunological responses in precision‐cut lung slices

**DOI:** 10.14814/phy2.15873

**Published:** 2023-11-23

**Authors:** B. Patlin, L. Schwerdtfeger, S. Tobet

**Affiliations:** ^1^ Department of Biochemistry and Molecular Biology Colorado State University Fort Collins Colorado USA; ^2^ Department of Neurology Harvard Medical School and Ann Romney Center for Neurologic Diseases, Brigham and Women's Hospital Boston Massachusetts USA; ^3^ School of Biomedical Engineering and Department of Biomedical Sciences Colorado State University Fort Collins Colorado USA

**Keywords:** calcitonin gene‐related peptide, neural fibers, precision‐cut lung slice, pulmonary neuroendocrine cells, surfactant

## Abstract

Organotypic lung slices, sometimes known as precision‐cut lung slices (PCLS), provide an environment in which numerous cell types and interactions can be maintained outside the body (ex vivo). PCLS were maintained ex vivo for up to a week and demonstrated health via the presence of neurons, maintenance of tissue morphology, synthesis of mucopolysaccharides, and minimal cell death. Multiple phenotypes of neuronal fibers were present in lung slices with varied size, caliber, and neurotransmitter immunoreactivity. Of the neuropeptides present in fibers, calcitonin gene‐related peptide (CGRP) was the most prevalent. Exposing PCLS to recombinant CGRP resulted in the proliferation and dispersion of CD19^+^ B cells in slices taken selectively from females. The number of granules containing immunoreactive (ir) surfactant protein C (SPC), which are representative of alveolar type 2 cells, increased in slices from females within 24 h of exposure to CGRP. Additionally, ir‐SPC granule size increased in slices from males and females across 48 h of exposure to CGRP. Exposure of PCLS to exogenous CGRP did not alter the number of solitary pulmonary neuroendocrine cells (PNEC) but did result in neuroendocrine bodies that had significantly more cells. Neuronal fiber numbers were unchanged based on ir‐peripherin; however, ir‐CGRP became non‐detectable in fibers while unchanged in PNECs. The effects of exogenous CGRP provide insight into innate immune and neuroendocrine responses in the lungs that may be partially regulated by neural fibers. The sex‐dependent nature of these changes may point to the basis for sex‐selective outcomes among respiratory diseases.

## Graphical abstract

Schematic of the precision cut lung slice model where lungs are removed from a mouse and slices are cultured in vitro. Cartoon representation shows calcitonin gene related peptide (CGRP) added to dishes resulted in a sex dependent increase in B cell and neuroendocrine body (NEB) cell populations and changes in surfactant protein C (SPC) containing granule size and number.

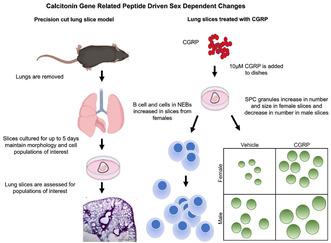

## 1 INTRODUCTION

The lungs are a primary surface of interaction with the external world—encountering pathogens, dirt, yeast, smoke, and other particulates in the thousands of liters of air a person breathes every day (Fang et al., 2020; Valavanidis et al., 2013). These particulates can cause respiratory damage, leading to pathologies including chronic obstructive pulmonary disease (COPD). Respiratory diseases like COPD are highly sex‐dependent (Barnes, 2016; Hong et al., 2016; Trigueros et al., 2019) and cause nearly four million deaths globally every year (Li, Cao, et al., 2020). While significant research efforts are devoted to studying the etiology of these diseases and pathways towards treatments, many unanswered questions about the nature of respiratory disease progression remain to be explored (Li, Cao, et al., 2020). Investigations of the diverse components of respiratory disease resistance and progression require methods that consider systems integration to account for the myriad cell–cell interactions involved in the disease etiology of the lung. In particular, the role of neural immune signaling at a local level requires further derivation. This study utilizes precision‐cut lung slices (PCLS) that maintain neural and immune elements inside the lungs, allowing investigation of complex neuroimmune signaling pathways (Blake et al., 2019).

A variety of cells in the lung produce neuroactive peptides such as calcitonin gene‐related peptide (CGRP), vasoactive intestinal peptide (VIP), and substance P. These neuropeptides regulate functions in the lung such as cell proliferation, nociception, and controlling airway homeostasis (Assas et al., 2014; Dakhama et al., 2004). CGRP is broadly implicated in neuroimmune axis functions of the lung. It plays a role in mucus secretion (Atanasova & Reznikov, 2018) and has been shown to limit the effects of some inflammatory cytokines (Nagashima et al., 2019). CGRP antagonists are being used to treat migraine (Assas, 2021; Benemei et al., 2009; Deen et al., 2017; Durham, 2006; Durham & Vause, 2010; Ray et al., 2021), and agonist effects are being evaluated in cancer (Dallmayer et al., 2019; Gutierrez & Boada, 2018; Höppener et al., 1987; Ostrovskaya et al., 2019; Zhang et al., 2020). Given the broad roles of CGRP in homeostasis and disease, it is important to understand the cell signaling pathways of this peptide in the lungs.

CGRP is a 37‐amino acid peptide with alpha and beta forms. While CGRPα and CGRPβ isoforms are nearly identical in structure—differing by only three amino acids—they are coded by separate genes. CGRPα is systemic, while CGRPβ is localized to the enteric nervous system (Russell et al., 2014). CGRPα is made from a gene splice variant of the gene for calcitonin, which is also known as procalcitonin—a peptide that can be used to identify bacterial pathogenesis through blood samples (Mazaheri et al., 2022). The alpha isoform is the most abundant manifestation of the peptide throughout the body, including in the (i.e., lungs), and has widespread peripheral effects, including vasculature constriction and involvement in pain (Russell et al., 2014). It is the form used for the current study. Commonly associated with C fibers, this peptide has receptors throughout the body. While normal levels of CGRP are around 1 μM in circulation, it can be released in response to inflammatory factors or capsaicin, increasing up to 10 μM in serum (Durham, 2006).

Pulmonary neuroendocrine cells (PNECs) are specialized epithelial cells that are innervated by CGRP immunoreactive neuronal fibers and contain neuropeptides (e.g., CGRP), amine hormones, and peptide hormones (Cutz, 1982). PNECs cluster and are subsequently referred to as neuroendocrine bodies (NEBs). PNECs and NEBs are commonly located near the intersection points of the airways and vasculature (Sui et al., 2018), which also contain germinal centers with a variety of resident immune cells, including B cells. PNEC behaviors have been incompletely characterized and are different in isolated versus clustered cells (Noguchi et al., 2020). Solitary PNECs are located sporadically throughout the airway epithelia and move in response to stimulation. Conversely, NEBs are usually located near airway junctions and do not move. PNECs and NEBs have been suggested to act as chemosensors (Gu et al., 2014). Although these cells are reportedly rare, there are several factors that stimulate population expansion or an increase in cell size (Mou et al., 2021), including hypoxia, lung injury, and disease (Shivaraju et al., 2021).

In the current study, lung‐specific interactions relevant to neural factors were examined in PCLS to investigate CGRP effects on alveolar type 2 (AT2) cells, CD19^+^ B cells, PNEC/NEB, and neuronals.

## 2 MATERIALS AND METHODS

### 2.1 Animals

Young adult male and female C57BL/6J mice transgenic for YFP expressed on the Thy‐1 promoter (JAX:003709) were used for all experiments (Feng et al., 2000; Tobet et al., 2003). Animals were housed in the Painter Center building and were cared for by Laboratory Animal Resources at Colorado State University. Cages were supplied with aspen bedding (autoclaved Sani‐chips; Harlan Teklad), tissues, and red igloo mouse houses. Mice were exposed to a 14:10 h light–dark cycle and had access to water and food ad libitum (no. 8640; Harlan Teklad). Animals were cared for and utilized according to Colorado State University IACUC protocol #1934.

### 2.2 Generation of PCLS

Adult mice were deeply anesthetized using isoflurane and killed via cervical dislocation. The thoracic cavity was opened by cutting through both sides of the ribs and then severing the diaphragm across the midline. Animals were perfused through the heart with 37°C phosphate buffered saline (PBS) for quantitative analyses or PBS containing 0.1 mg/mL FITC to aid visualization of the vasculature for qualitative examination and imaging. Hearts were removed, and the thymus and salivary glands were dissected away under an Olympus SZX7 dissecting scope until the trachea was clearly visible. The trachea was partially perforated so that a 20‐gauge needle could be inserted. The lungs were inflated using 1 mL of 40°C 2% low melting point agarose (Gold Biotechnology, St. Louis, MO) in MQH_2_O (Milli‐Q ultrapure water) until the lung was visibly inflated to the edge of the lobe without rupturing. Lungs were removed and immediately placed in ice‐cold Krebs. Using the Olympus SZX7 dissecting scope, the trachea and blood clots were dissected from the exterior of the lung, and lobes were separated. Single lobes of the lung were then placed in ~40°C 8% low melting point agarose in MQH_2_O at 4°C for 4 min to permit the agarose to polymerize and enable cutting of 250 μm thick slices using a vibrating microtome (VT1000S; Leica Microsystems, Wetzlar, Germany). Slices were collected into 5 mL hibernate media (Life Technologies, Grand Island, NY) in 60 mm dishes (Corning, Corning, NY) at 4°C for at least 15 min; hibernate was then removed and replaced with 5 mL of adult neurobasal culture media (ANB; Life Technologies) with 2% B27 supplement (Life Technologies) for 35 min at 37°C to permit slices to regain physiological temperature. This medium was serum‐free to increase the reproducibility of experiments (Schwerdtfeger et al., 2019), and antibiotics were omitted from the media to promote the maintenance of the lung microbiota and translational relevance. Slices were relocated to 35‐mm plastic bottom culture dishes (Corning and Corning Falcon, Corning, NY) sans media for 15 min at 37°C and 100% humidity to promote adherence to the dish. Slices were then covered with a thin layer of collagen solution (vol/vol: 10.4% 10× MEM, 1.9% PS, 4.2% sodium bicarbonate, and 83.5% collagen (PureCol; Inamed, Fremond, CA)) for 10 min at 37°C before 0.8 mL of adult neurobasal media with 2% B27 supplement was added to each dish to create a culture with an air–liquid interface. The media was changed daily throughout the culture period.

### 2.3 Neuropeptide treatment

PCLS were treated with 10 μM CGRPα peptide (015‐09; Phoenix Pharmaceuticals) for 24 h, or 48 h. Dosage was based on levels of CGRP released from trigeminal neurons in response to different stimuli (Durham, 2006). To confirm dosage, slices were treated with 1 μM or 10 μM and compared for B‐cell response. Ten micrometer CGRP addition generated a larger B‐cell response and thus was used for all experiments. At the end of the incubation/treatment period, slices were fixed in 4% paraformaldehyde for 15 min and washed three times for 1–2 min with PBS.

### 2.4 Biochemical analyses

To qualitatively assess cell death, lung slices were visualized after incubating for 10 min in acridine orange (AO; A1301, Invitrogen, Thermo Fisher Scientific) at 1 μL per 0.8 mL of media (2 μM), followed by 3× 30‐min washes with 1 mL of media (Byvaltsev et al., 2019). In a subset of slices during the procedure development phase, health was confirmed by live treatment with AO and imaging of tissue using a Texas Red filter set (fluorescent emission 656) to visualize cells with permeable nuclei. Protocol and methodological development were performed until less than ~10% of the cells visualized had permeable nuclei (data not shown). To assess mucopolysaccharide synthesis in lung epithelia, slices were treated with tetraacetylated‐N‐Azidoacetylgalactosamine (GalNAz; C33365, Thermo Fisher) after 0, 24, or 48 h of culture and incubated for an additional 24 h after addition of GalNAz. Slices were fixed using 4% paraformaldehyde and reacted with the fluorophore‐tagged alkyne, Dibenzocyclooctyne‐Cy3 (DBCO‐Cy3; 2 μM; Sigma‐Aldrich, St. Louis, MO) to produce fluorescence (Schwerdtfeger et al., 2016). To assess qualitative cell proliferation, slices were treated with 4 μL of 10 μM 5‐ethynyl‐2′‐deoxyuridine (EdU; Invitrogen, Eugene, OR) per 0.8 mL of media after 24 h of culture. At 48 h, EdU containing media was removed from slices before fixation with 4% paraformaldehyde. Slices were then washed in PBS for 30 min, followed by 30 min in glycine (Fisher Scientific, Pittsburgh, PA), and 3 more PBS washes to remove any unreacted 4% paraformaldehyde. Slices were blocked in 3% bovine serum albumin buffer (BSA; Lampire Biological, Pipersville, PA) and 0.5% Triton X‐100 (Tx; 42235‐5000, ACROS) for 2 h, followed by two washes with a 3% BSA solution for 10 min each. A click‐IT reaction was then performed on the slices using a click‐IT cocktail (click‐IT Reaction Buffer, CuSO4, Alexa‐Fluor azide, reaction buffer additive, Invitrogen) for 2 h. Slices were further washed three times in 3% BSA buffer and 0.02% Tx for 30 min each and left overnight in a 3% BSA solution. Samples were mounted on slides and cover‐slipped using Aqua‐Poly/Mount (Polysciences, Warrington, PA) for fluorescent imaging by confocal microscopy (a representative image is shown in Figure S8) (Schwerdtfeger et al., 2016).

### 2.5 Immunohistochemical analyses

Slices were fixed for 15 min in 4% paraformaldehyde at room temperature and then washed in 0.5 M PBS (pH 7.4) prior to whole‐mount immunohistochemistry (250 μm thick). Slices were incubated at 4°C in 1% sodium borohydride for 2 h. They were washed in PBS for 10 min prior to incubation in a blocking solution comprised of PBS with 5% normal goat serum (NGS; Lampire Biological, Pipersville, PA), 3% hydrogen peroxide, and 0.3% Tx for 2 h with a change of solution at 1 h. Slices were then incubated in a primary antisera (see Table 1) solution containing 5% NGS, 0.3% Tx, and primary antibodies at various concentrations for 5 days. Then, slices were washed at 4°C in a 1% NGS, 0.2% Tx, and PBS solution four times for 30 min per wash. Slices were then incubated for 24 h at 4°C in fluorescent conjugated secondary antisera. After 24 h, samples were washed four times for 30 min each in a 0.02% Tx PBS solution before being mounted on slides and covered slipped with Aqua‐Poly/Mount (Polysciences, Warrington, PA).

**TABLE 1:** Antibodies used for immunohistochemistry.

Primary antibodies	RRID	Source	Secondary antibodies	RRID	Source	Marker for
CD19	AB_467151	14‐0199‐82 eBioscience	Cy3 anti‐rat 1:500	AB_2338253	112‐166‐003 Jackson ImmunoResearch	B cells
CD79	AB_962641	NB10064347 Novus Biologicals	Cy3 anti‐rabbit 1:500	AB_2338008	111‐166‐045 Jackson ImmunoResearch	B cells
Peripherin	AB_90725	Ab1530 Arc Bio	Cy3 anti‐rabbit 1:500	AB_2338008	111‐166‐045 Jackson ImmunoResearch	Type 3 intermediate filament protein
SPC	AB_91588	Ab3786 EMD Millipore	Cy3 anti‐rabbit 1:500	AB_2338008	111‐166‐045 Jackson ImmunoResearch	Surfactant protein C (SPC)
VIP	AB_572270	20077 Immunostar	Cy3 anti‐rabbit 1:500	AB_2338008	111‐166‐045 Jackson ImmunoResearch	Vasoactive intestinal peptide
CGRP	AB_572217	24112 Immunostar	Cy3 anti‐rabbit 1:500	AB_2338008	111–166‐045 Jackson ImmunoResearch	Calcitonin gene‐related peptide (CGRP)
ChAT	AB_196848 4	NBP1‐ 30052 Novus Biologicals	Cy3 anti‐goat 1:500	AB_2340880	805‐165‐180 Jackson ImmunoResearch	Choline acetyl transferase
Substance P	AB_572266	20064 Immunostar	Cy3 anti‐ rabbit 1:500	AB_2338008	111‐166‐045 Jackson ImmunoResearch	Substance P peptide

### 2.6 Microscopy and image analysis

Slices were imaged by confocal microscopy using a Zeiss LSM 880 confocal microscope with an Axiocam 503 mono camera (Carl Zeiss, Inc., Thornton, NY) after fixation or a Nikon TE2000‐U inverted microscope for live brightfield or epifluorescence imaging. TE2000‐U images were taken using a 20× Plan‐Apo objective lens. For each set of treatment groups (24 h CGRP or 48 h CGRP exposure, or 24, 72, and 120 h vehicle exposure), samples were collected at the same time under the same conditions. Confocal imaging was performed for immunohistochemical analysis of all slices. At least three mice were used for each experiment to account for biological variability. Single‐plane confocal images were taken using 20× and 40× objective lenses (plan‐fluor). At least one PCLS was analyzed in each analysis. All images were selected to contain the maximal number of puncta of interest with 0 overlap in sets of 12 images. Analyses were considered per slice, and *n* values are reported as the number of slices analyzed. Live imaging was performed for GalNAz, acridine orange, and slices from FITC‐perfused lungs. Fixation eliminated fluorescence generated by the Thy‐1 promoter driving YFP. The investigator conducting all quantitative analyses was blinded to the treatment group.

### 2.7 Quantitative analyses

Analyses were performed for the number and/or size of each labeled entity using NIH Fiji (v.2.9.0). All regions of interest (ROIs) were 426–426 μm. The area of immunoreactive SPC was calculated in a series of 12 regions of interest from single‐plane images for each experimental value. Neuronal fibers (CGRP‐ir and peripherin‐ir) were counted by area per ROI and by area of fibers per slice. Fiber ROIs were localized to bronchioles, as bronchioles were the primary site of innervation, and 12 ROIs per slice were assessed per experimental data point. CGRP‐ir cells (PNECs and NEBs) were counted manually without computer assistance. They were identified in sets of 12 ROIs in a single plane from each slice for each experimental value. CD19^+^ labeled cells were analyzed in 12 ROI's selected for the presence of CD19^+^ cells for each experimental slice data point. All measurements except those for PNECs and NEBs recorded included cell counts, area, mean, and standard deviation.

### 2.8 Statistics

Statistical analysis was performed using Prism 9.4.0 (GraphPad) and Microsoft Excel software. A two‐way ANOVA (sex x time of CGRP) was performed for males versus females for 24 h CGRP, 48 h CGRP, 24, 72, and 120 h vehicle groups for all analyses. SPCs, or CD19^+^ B cells, were quantified by size and number, and CGRP, or peripherin, by total fiber area and fiber area/slice. Post‐hoc testing used a multiple comparison Fisher's LSD test, and all data is presented as mean ± standard deviation. *p* < 0.05 was considered significant, and for the figures, it was represented with one symbol, *p* < 0.01 was represented with two symbols, and *p* < 0.001 was represented with three symbols.

### 2.9 Tissue health and criteria for data inclusion

Only data generated from tissue that was qualified as healthy was utilized in this study. The health of lung slices—maintained ex vivo for 5 days—was demonstrated using a variety of methods during the optimization process before experiments were performed (Figures S3–S8). Cells lining the bronchioles continued to synthesize mucopolysaccharides ex vivo, similar to in vivo (Figure S1). GalNaz Click‐chemistry allowed for visualization of newly incorporated mucin‐type O‐linked glycoproteins around bronchioles. Ciliary beating was confirmed in slices by visual observation (data not shown). Lung slices (250 μm) maintained structural morphology for at least 5 days ex vivo, as shown in representative fluorescent images (Figure S2) and using the histological stains thionin and aldehyde fuchsin (data not shown).

Neuronal fiber integrity was also used as an indicator of health. Such fibers were revealed based on the immunohistochemical detection of peripherin (Figure S3a), choline acetyl transferase (Figure S3b), substance P (Figure S3c), vasoactive intestinal peptide (Figure S3d), and CGRP‐ir (Figure S3e) fiber populations. CGRP‐ir fibers had the highest abundance, excluding peripherin, which is a comprehensive marker for peripheral fibers.

## 3 RESULTS

**FIGURE 1:**
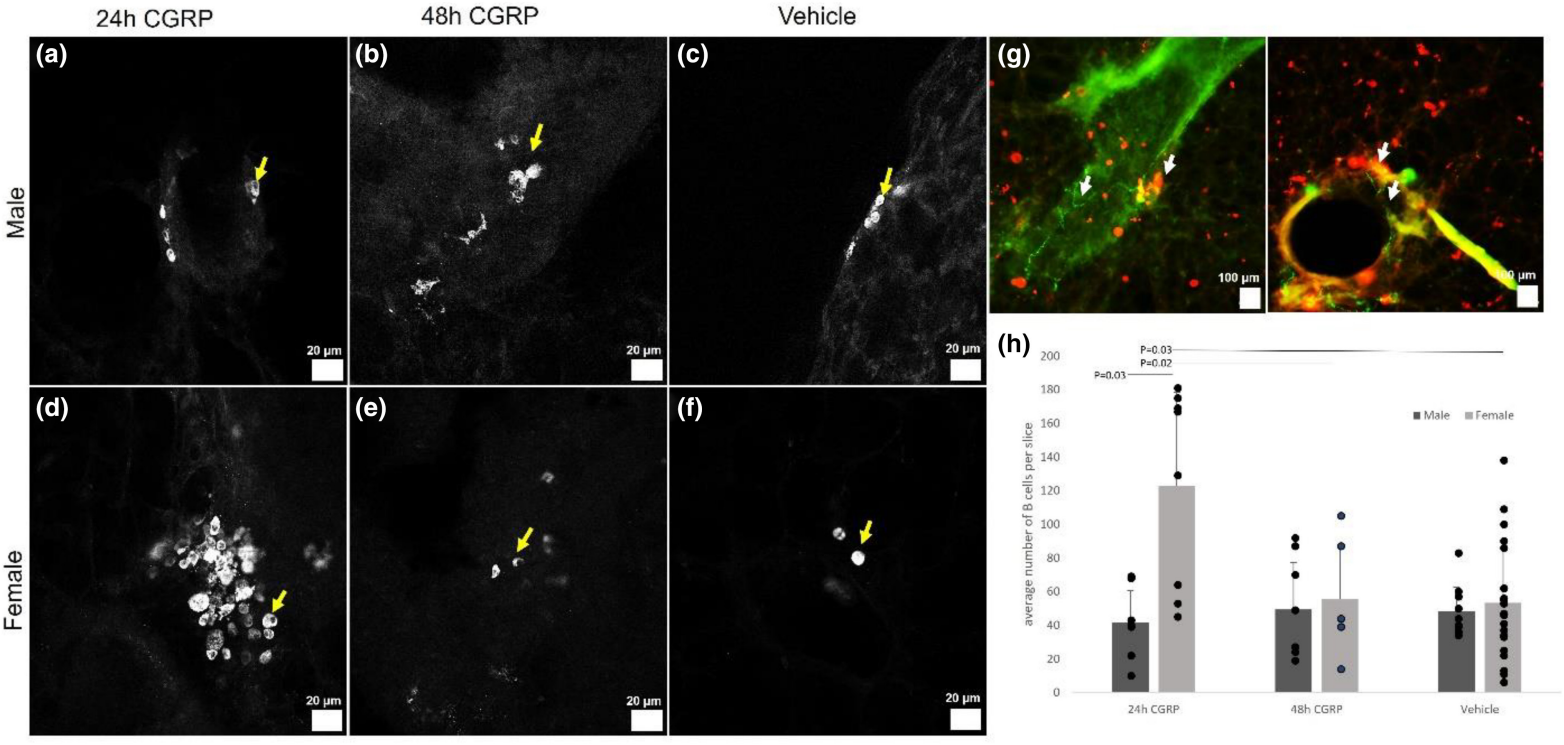
The number of CD19^+^ immunoreactive B cells was greater in slices from females but not males upon treatment with exogenous calcitonin gene‐related peptide (CGRP). (a–c) Male PCLS after exposure to CGRP for 24 or 48 h compared to vehicle. (d–f) Female PCLS after exposure to CGRP for 24 or 48 h compared to the vehicle. (g) CGRP fibers (green) colocalized with B cells (red). (h) B cell numbers were significantly greater in slices taken from females after 24 h of exposure to CGRP compared to the vehicle and greater than B cell numbers in slices from males. No difference was seen at 48 h compared to the vehicle, regardless of the sex of the slice origin. B cell numbers in slices from males did not differ significantly with CGRP treatment. Yellow and white arrows indicate B cells. Dark gray indicates male, light gray indicates female, and bars are paired as 24 h of treatment with CGRP, 48 h of treatment with CGRP, or vehicle. Twenty‐four hours CGRP‐male *n* = 5 mice/8 PCLS; 24 h CGRP‐female *n* = 5 mice/9 PCLS; 48 h CGRP‐male *n* = 4 mice/8 PCLS; 48 h CGRP‐female *n* = 4 mice/6 PCLS; vehicle‐male *n* = 6 mice/9 PCLS; vehicle‐female *n* = 12 mice/24 PCLS. Scale bars are 20 μm in (a–f) and 100 μm in (g).

A striking modulatory effect of the immune axis was observed when slices were given CGRP‐containing media. Exposing PCLS to CGRP resulted in a 120% greater number of CD19^+^ B cells in slices from female mice (Figure 1d,f; *p* = 0.03; *n* = 9 24 h CGRP vs. 24 vehicle) but not male mice (Figure 1a,c; *p* = 0.97; *n* = 8 24 h CGRP vs. 9 vehicle) within 24 h of treatment with 10 μM CGRP. Within 48 h of 10 μM CGRP exposure, the CD19^+^ B‐cell population reverted to numbers comparable to vehicle‐treated controls across 5 days ex vivo of vehicle treatment in females (Figure 1f; *p* = 0.02; *n* = 6 48 h CGRP vs. 24 vehicle). Consistent with these observations, CD19^+^ B cells were regularly found alongside CGRP fibers (Figure 1g). In slices from males, there were no changes in CD19^+^ B‐cell populations compared with vehicle (Figure 1b,c; *p* = 0.84; *n* = 8 24 h CGRP vs. 9 vehicle). There were no significant differences in CD19^+^ B cell numbers in slices from males (*n* = 8) and females (*n* = 9) treated with CGRP at 48 h (Figure 1a–c compared to Figure 1d–f; *p* = 0.94).

**FIGURE 2:**
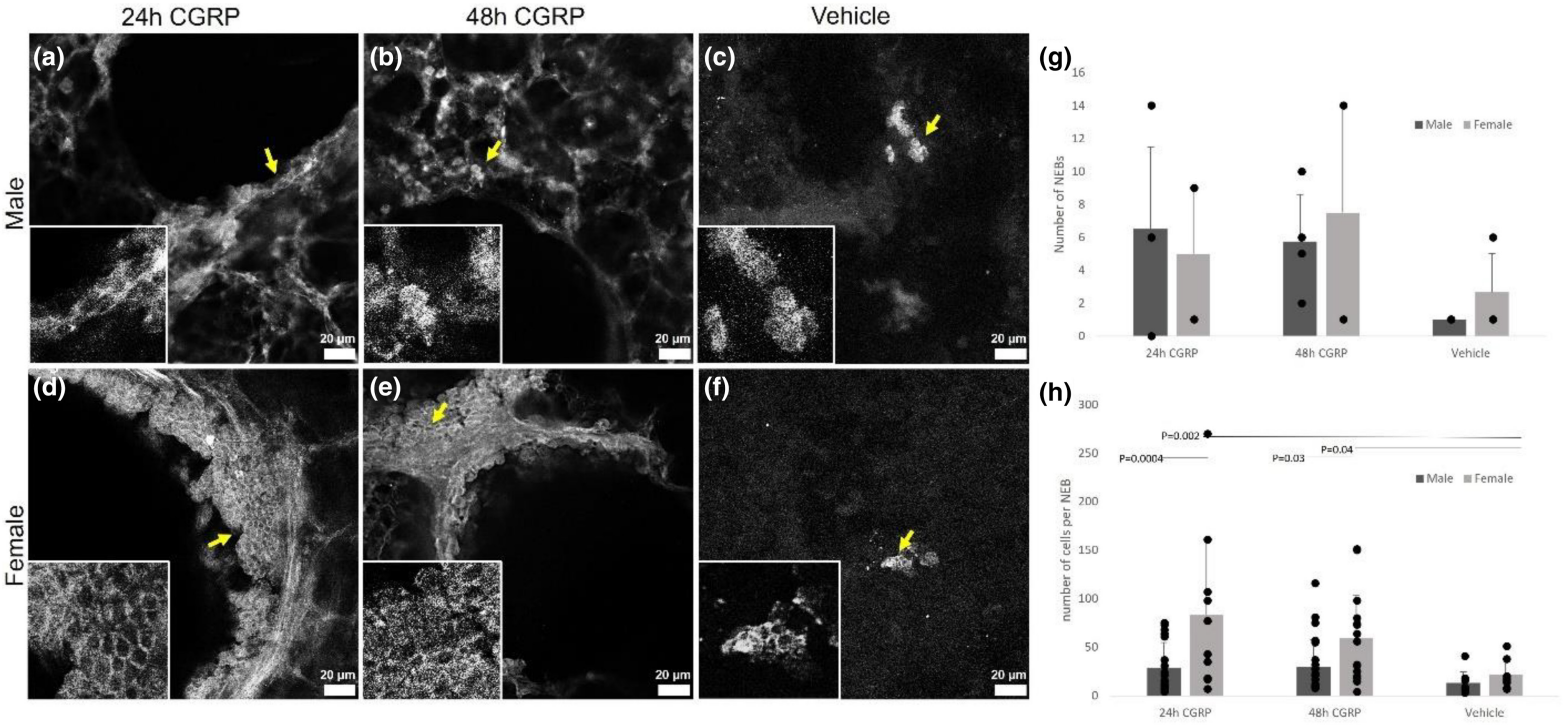
The number of neuroendocrine bodies (NEBs) did not change upon treatment with calcitonin gene‐related peptide (CGRP) over 24 or 48 h of CGRP treatment, and there were no sex differences in the number of NEBs. The size of NEBs, measured by the number of cells per NEB (H), was greater in slices from females after 24 or 48 h of CGRP exposure compared to the vehicle. NEBs in slices from females contained more CGRP^+^ cells than in slices from males when exposed to CGRP for 24 or 48 h. There were no significant differences in the number of cells/NEB in slices from males at any timepoint or treatment. (a–c) Male representative images of NEBs. (d–f) Female representative images of NEBs. (g) number of NEBs. (h) Average number of cells in a NEB. The yellow arrow indicates the location of the inserts at higher magnification. Dark gray indicates male, light gray indicates female, and bars are paired as 24 h of treatment with CGRP, 48 h of treatment with CGRP, or vehicle. Twenty‐four hours CGRP‐male *n* = 3 mice/4 PCLS (G)/24 NEBs (H); 24 h CGRP‐female *n* = 3/4 PCLS(G)/10 NEBs (H); 48 h CGRP‐male *n* = 4/4 PCLS (G)/23NEBs (H); 48 h CGRP‐female *n* = 3/4 PCLS (G)/15 NEBs (H); vehicle‐male *n* = 3/3 PCLS(G)/7 NEBs (H); vehicle‐female *n* = 3/3 PCLS (G)/7 NEBs (H). Scale bars are 20 μm.

NEBs in slices from females were larger after treatment with CGRP. The number of cells per NEB in slices from females was over four times greater after 24 h of CGRP exposure (*p* = 0.0003; *n* = 5) (Figure 2d,f) and three times greater after 48 h of CGRP exposure (*p* = 0.04; Figure 2e,f; *n* = 5) compared to vehicle (*n* = 20). The number of cells per NEB in slices from males was similar after 24 h (*p* = 0.33; Figure 2a,c; *n* = 6 24 h CGRP vs. 12 vehicle) or 48 h of CGRP exposure compared to vehicle (*p* = 0.31; Figure 2b,c; *n* = 6 48 h CGRP vs. 12 vehicle). The number of cells per NEB in slices from female lungs with 24 h of CGRP exposure was almost three times greater than the number of cells per NEB in a 24 h CGRP‐exposed slice from male lungs (*p* = 0.0004; Figure 2a,d; *n* = 5 female vs. 6 male). The number of cells per NEB in 48 h CGRP‐exposed slices from females was almost two times greater than in similar slices from males (*p* = 0.03; Figure 2b,e; *n* = 5 female vs. 6 male). There was no difference in solitary PNECs with treatment or over time. In both vehicle and CGRP‐treated slices, the number of PNECs ranged from 10 to 25 cells. Because NEB size increased by up to 60 cells per NEB when treated with CGRP with no change in the number of solitary PNECs, some proliferation occurred. Cell migration may also have contributed to the increase in the number of cells per NEB.

**FIGURE 3:**
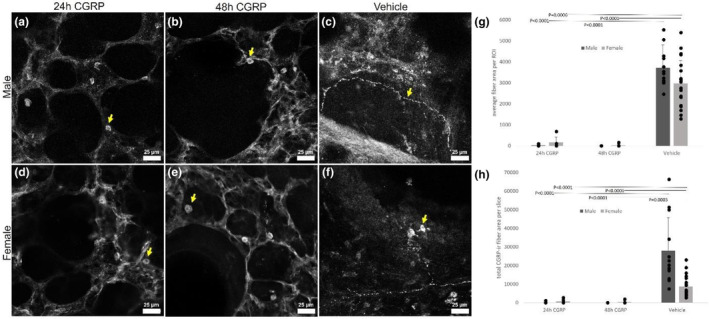
Fibers containing immunoreactive calcitonin‐gene related peptide (CGRP‐ir) were not detected after exposure to exogenous CGRP. By contrast, pulmonary neuroendocrine cells were still defined by CGRP‐ir. (a–c) Lung slices from males containing ir‐CGRP and after exposure to exogenous CGRP for 24‐ or 48 h compared to the vehicle. (d–f) Lung slices from females containing ir‐CGRP and after exposure to exogenous CGRP for 24‐ or 48 h compared to vehicle. (g) The average CGRP‐ir area per ROI was greatly decreased in slices treated with CGRP over 24 and 48 h. (h) The total area of CGRP in a slice dropped to negligible levels at 24 and 48 h of treatment with CGRP, and there was more CGRP‐ir in male slices than female slices. Yellow arrows in (a–b) and (d–e) point to PNECs; yellow arrows in (c) and (f) indicate fibers. Dark gray indicates male, light gray indicates female, and bars are paired as 24 h of treatment with CGRP, 48 h of treatment with CGRP, or vehicle. Twenty‐four hour CGRP‐male *n* = 4 mice/6 PCLS; 24 h CGRP‐female *n* = 4 mice/5 PCLS; 48 h CGRP‐male *n* = 4 mice/6 PCLS; 48 h CGRP‐female *n* = 5 mice/5 PCLS; vehicle‐male *n* = 9 mice/12 PCLS; vehicle‐female *n* = 11 mice/20 PCLS. Scale bars are 20 μm.

**FIGURE 4:**
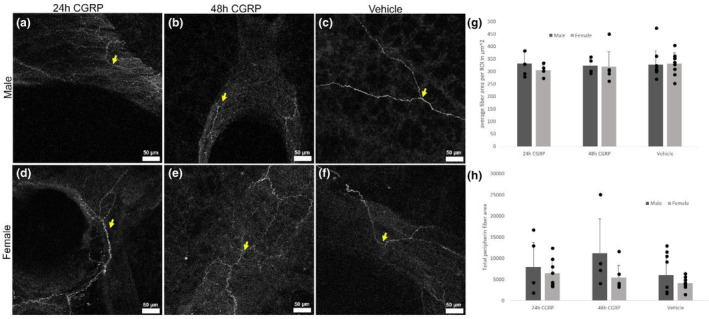
Fibers containing immunoreactive peripherin (ir) did not change after exposure to exogenous CGRP. (a–c) Lung slices from males examined for ir‐peripherin (yellow arrows) exposed to exogenous CGRP after 24 h or 48 h compared to vehicle. (d–f) Lung slices from females examined for ir‐peripherin (yellow arrows) exposed to exogenous CGRP after 24 h or 48 h compared to vehicle. (g) Quantification of ir‐peripherin fiber areas/region of interest (ROI) after 24 or 48 h of vehicle versus CGRP exposure in slices from males and females. (h) No change in total peripherin‐ir fibers was found in 24 h or 48 h CGRP‐treated slices. Dark gray indicates male, light gray indicates female, and bars are paired as 24 h of treatment with CGRP, 48 h of treatment with CGRP, or vehicle. Twenty‐four hour CGRP‐male *n* = 3 mice/5 PCLS; 24 h CGRP‐female *n* = 6 mice/8 PCLS; 48 h CGRP‐male *n* = 3 mice/4 PCLS; 48 h CGRP‐female *n* = 6 mice/7 PCLS; vehicle‐male *n* = 7 mice/9 PCLS; vehicle‐female *n* = 8 mice/13 PCLS. Scale bars are 50 μm.

Exposing lung slices to exogenous CGRP virtually eliminated ir‐CGRP in fibers ex vivo. The average combined area of CGRP‐ir fibers per ROI approached zero in 10 μM CGRP‐exposed tissue over 24 h and 48 h compared to vehicle (Figure 3c,f) for slices from both males (Figure 3a–c; *p* < 0.0001; *n* = 4) and females (Figure 3d–f; *p* < 0.0007; *n* = 8). However, when treated with 10 μM CGRP for 24 h (Figure 4a,d) or 48 h (Figure 4b, e), the average area of peripherin fibers per ROI was similar to vehicle (Figure 4c, f; *p* = 0.62; *n* = 13) and not different between slices from male (Figure 4a–c; *n* = 7) and female (Figure 4d–f) PCLS (*p* = 0.79; *n* = 9).

**FIGURE 5:**
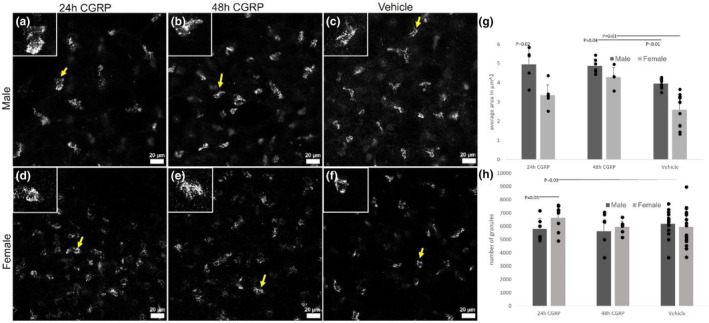
The area of granules containing immunoreactive surfactant C (ir‐SPC) was significantly greater in slices from females after exposure to exogenous CGRP after 48 h. (a–c) Lung slices from males were examined for ir‐SPC after exposure to exogenous CGRP for 24‐ or 48 h compared to vehicle. (d–f) Lung slices from females were examined for ir‐SPC after exposure to exogenous CGRP for 24‐ or 48 h compared to vehicle. (g) In slices from females, ir‐SPC granules were significantly smaller than those in slices from males after 24 h of exogenous CGRP treatment and in the vehicle. The size of granules was greater in males and females exposed to CGRP for 48 h. (h) The number of ir‐SPC granules was greater in females after 24 h of CGRP exposure compared to vehicles. Dark gray indicates male, light gray indicates female, and bars are paired as 24 h of treatment with CGRP, 48 h of treatment with CGRP, or vehicle. Twenty‐four hour CGRP‐male *n* = 3 mice/6 PCLS; 24 h CGRP‐female *n* = 4 mice/8 PCLS; 48 h CGRP‐male *n* = 3 mice/7 PCLS; 48 h CGRP‐female *n* = 5/7 PCLS; vehicle‐male *n* = 9 mice/18 PCLS; vehicle‐female *n* = 9 mice/21 PCLS. Scale bars are 10 μm.

Type 2 pneumocyte populations indicated by ir‐SPC were significantly greater in area after 48 h of 10 μM CGRP exposure compared to vehicle for both male (*p* = 0.01; *n* = 7) and female (*p* = 0.0008; *n* = 7) PCLS (Figure 5g). The size of granules containing ir‐SPC in slices from females was significantly smaller than in males at 24 h of 10 μM CGRP treatment (*p* = 0.017; *n* = 8 female, 6 male) and in the vehicle (*p* = 0.0004; *n* = 21 female, 18 male). The number of granules in slices from females was significantly increased at 24 h of 10 μM CGRP exposure compared to vehicle (*p* = 0.031; *n* = 8 24 h CGRP, 21 vehicle). The number of granules in slices from females was significantly greater than the number in male slices when treated for 24 h with 10 μM CGRP (Figure 5h; *p* = 0.027; *n* = 8 female, 6 male). Reference images for SPC‐labeled male (Figure 5a–c) and female (Figure 5d–f) PCLS are provided for 24 h CGRP (Figure 5a,d), 48 h CGRP (Figure 5b,e), and vehicle (Figure 5c,f) exposures.

## 4 DISCUSSION

Exposure of lung tissue to CGRP resulted in several immunological, physiological, and neural changes in lung tissue. These changes in local neural and immune signaling pathways were observable using an ex vivo PCLS model that eliminated influences from other organs. The current study demonstrated changes in B‐cell populations, the number of surfactant granules, and NEB size increases in slices from females after 24 h of exposure to 10 μM CGRP. In parallel to these findings, lung slices from females contained a smaller CGRP‐ir fiber population than in slices from males, and these CGRP‐ir fiber populations disappeared after treatment with CGRP in slices from both sexes. Sex‐related changes caused by CGRP exposure ex vivo were consistent with sex differences in respiratory disease responses (Jacobsen & Klein, 2021; Klein & Flanagan, 2016; Ursin & Klein, 2021). The disappearance of fibers containing ir‐CGRP, in conjunction with the retention of those containing ir‐peripherin, may indicate an efflux of CGRP and subsequent lack of detectable signal from fibers rather than a loss of fibers themselves. The current study addressed the role of CGRP neuronal signaling in immune and epithelial components using PCLS maintained ex vivo for 5 days. While in vivo studies have previously reported a diversity of neuropeptide‐producing fibers in the lung, PCLS have rarely been used when considering them (Schlepütz et al., 2012). CGRP was detected in neuronal fibers and in PNECs, a unique epithelial endocrine cell population, consistent with previous literature (Cutz, 1982; Mou et al., 2021). When exogenous CGRP was added to lung slices, changes in several lung cell characteristics depended upon the sex of the mice.

A major finding of the current study is that exposure to exogenous CGRP resulted in a greater number of CD19^+^ B cells in slices obtained from females but not males. This result was potentially driven by the trend of females towards elevated humoral immunity at baseline, including increased adaptive immune cell expression and antigen responses (Fink & Klein, 2018). These CD19^+^ B cells were found in germinal centers in PCLS maintained ex vivo across a 5‐day period, providing evidence of bronchiolar associated lymphoid tissue (BALT) maintenance and allowing for exploration of resident immune cell populations in this model. Exogenous CGRP addition resulted in higher numbers of CD19^+^ B cells throughout female lung slices, not just in BALT. Cell dispersion was likely responsible for the exit of B cells from germinal centers/BALT located primarily in arteriole bronchiole junctions. Such B‐cell movements and behaviors are similar to the propagation of airway epithelial cells in that they respond to injury caused by inhalants or lipopolysaccharides (Oslund et al., 2009; Zhou et al., 2013). Because of the similarities between extracellular CGRP exposure and innate pulmonary responses to injury or infection, it may be possible that CGRP release is a key component of lung tissue repair processes. This prediction is further substantiated by the increase in serum CGRP in response to capsaicin and LPS (Durham, 2006). The static nature of B‐cell anatomy and localization over 5 days without treatments suggests that damage caused to tissue by slicing did not result in any significant immune responses and thereby minimal release of cytokines that would otherwise be released from damaged cells (Henjakovic et al., 2008). Other factors that cause CD19^+^ B‐cell proliferation in conjunction with cytokine signaling are antigen binding or stimulation from an activated helper T cell (Janeway Jr., 2001; Ollila & Vihinen, 2005). However, there was no evidence of T‐cell proliferation in slices based on CD3 immunoreactive T cells (data not shown).

CGRP exposure increased the number of cells in NEBs, and the quantity of NEBs in tissue increased in slices derived from females but not males, potentially driving the observed downstream immune cell population changes. PNECs move to form NEBs, but clustered cells within NEBs can also proliferate, have been implicated in lung cancer, and exhibit stem cell functions (Chen et al., 2019; Gutierrez & Boada, 2018; Ouadah et al., 2019). PNECs are known to increase in response to hypoxia and viral disease (Benson et al., 2013; Chen et al., 2019; Kawanami et al., 2009; Shivaraju et al., 2021). Yet, the number of PNECs and PNECs per NEB in control tissue were comparable to previously reported values. In CGRP‐treated tissue, the data showed similar numbers of free PNECs compared to control tissue, but an increase in the number of PNECs per NEB in females (Branchfield et al., 2016; Ouadah et al., 2019), pointing towards the larger cell counts being indicative of proliferation. PNECs can act as chemosensors that release neuropeptides and hormones, which are known to cause various changes to lung physiology, tissue remodeling, and the activation of the immune response (Cutz, 1982; Gu et al., 2014; Kaczyńska et al., 2018; Song et al., 2012). Stimuli that are correlated with an increase in NEB size, such as capsaicin exposure or bacterial infection, also increase the amount of serum and tissue CGRP (Bigal et al., 2015; Jean et al., 2022; Russell et al., 2014; Sui et al., 2018), consistent with the CGRP‐induced NEB size increase observed in the current study. Given the colocalization of CGRP fibers with neuroendocrine relevant cells containing neuropeptides and hormones, PNECs and NEBs may act to facilitate the observed neural‐driven immune response (Carey, Card, Voltz, Arbes Jr, et al., 2007; Noguchi et al., 2020) that was sex‐dependent in lung slices.

In conjunction with the changes caused by CGRP to immune and neuroendocrine cell populations, ex vivo CGRP addition resulted in the virtual elimination of neuronal fibers containing ir‐CGRP. To test whether CGRP exposure resulted in the removal of fibers carrying ir‐CGRP or whether the exposure simply caused the release of ir‐CGRP with fibers still present, a more general marker for peripheral neuronal fibers was examined. Exposure to CGRP did not reduce the quantity of peripherin‐ir fibers after 24 h or 48 h ex vivo. Therefore, it is unlikely that the previously ir‐CGRP fibers were eliminated from the slice via degradation. Instead, this disappearance may suggest a positive feedback loop of CGRP release from CGRP‐ir fibers upon interaction with the peptide, meaning that CGRP may be released when CGRP is externally present (e.g., pre‐“synaptic” receptors). By contrast, ir‐CGRP was not lost from PNECs or NEBs after treatment with exogenous CGRP, and an increase in PNEC or NEB fluorescent intensity was not obvious. The retention of ir‐CGRP in PNECs versus neuronal fibers may indicate a difference in the presence of a receptor across cell types.

Ex vivo treatment with CGRP increased SPC‐ir granule size and number, suggesting an effect on AT2 cell quantity and function. While CGRP has previously been shown to induce epithelial cell proliferation in cell lines, changes in SPC granule number and size have rarely been reported in PCLS (Dakhama et al., 2004; Fu et al., 2010; Kawanami et al., 2009; Li, Cohen, et al., 2020; Oslund et al., 2009). The change in SPC immunoreactivity in the tissue may indicate a change in tissue alveolar cell coverage because a greater number of observed granules and AT2 cells will result in less space for alveolar type 1 (AT1) projections. Comparatively, the increase in average size of granules indicates a higher level of SPC production and potentially secretion. The pneumocyte populations consist of AT1 and AT2 cells, which are responsible for air exchange and lung structure, respectively. The ratio of these two cell types is dynamic. Both AT1 and AT2 cells have been reported to transdifferentiate into the other under certain conditions to maintain homeostatic activity in the lung (Jain et al., 2015; Jansing et al., 2017; Rosenblum et al., 1998; Wang et al., 2018). The observed change in surfactant production and AT2 cells may suggest that CGRP exposure plays a role in the re‐epithelialization process in ex vivo slices, which has been shown in several other PCLS models relating primarily to idiopathic pulmonary fibrosis (Kiener et al., 2021; Ptasinski et al., 2021).

Although innervation of the lung has been explored in the past, it has only been minimally studied in a PCLS model. This study demonstrates the potential of PCLS as a model for investigating peripheral neuroimmune interactions. The nervous innervation of the lung is implicated in various defensive mechanisms against disease utilizing neuropeptide release. Neuronal exposure to neuropeptides may result in the release of diverse neuropeptides, resulting in complex reactions. For example, one known interaction between substance P, VIP, and CGRP can modulate inflammatory reactions such as mucus secretion in the lung (Atanasova & Reznikov, 2018; Chang et al., 2015). VIP, substance P, and CGRP are all located around the airways and maintain this vicinity in the PCLS utilized in these experiments. Few studies use PCLS models to study neural influences over lung physiology, and studies that do mostly consider airway smooth muscle regulation by respiratory neural components but not immune cells (Bai et al., 2022; Kummer et al., 2006; Lam et al., 2019; Schlepütz et al., 2012; van den Berg et al., 2021). Studies that have examined the roles of neuropeptides have either utilized electric field stimulation or explored the role of substance P in relation to MUC5AC expression and airway contraction (Regal & Johnson, 1983; Sponchiado et al., 2021; Springer & Fischer, 2003; Voedisch et al., 2012). Ex vivo models allow for the direct introduction of stimuli and the observation of anatomically localized responses. In the current study, neuronal fibers and immunoreactive neuropeptides were maintained ex vivo for over 5 days, making the model more complete and allowing a longer experimental time than some PCLS models. The current PCLS protocol allowed for up to 60 parallel slices from the same mouse, providing significant differences within mouse controls. This allowed the consideration of differences caused by environmental stressors, differential microbiota, and other factors that may be variable between mice.

Sex differences in immune responses and mortality to respiratory diseases are found across species (Klein & Flanagan, 2016). Respiratory inflammatory diseases are more common in females compared to males; however, males are more susceptible to diseases such as COVID‐19 and pneumonia (Chamekh et al., 2017; Klein et al., 2001). Patients presenting with similar symptoms of the same age and fitness can have drastically different outcomes depending upon their sex (Hong et al., 2016; Kautzky‐Willer et al., 2022; McNicholas et al., 2019; Tejpal et al., 2021; Trigueros et al., 2019; Ursin & Klein, 2021). In the lung, sex‐dependent neural differences include changes in acetylcholine release and neuromuscular function, regulation of airway constriction, ciliary clearance, and mucus secretion (Carey, Card, Voltz, Germolec, et al., 2007; Dominelli & Molgat‐Seon, 2022; Fuseini & Newcomb, 2017; Han et al., 2018; LoMauro & Aliverti, 2018; Townsend et al., 2012). These sex differences are influenced by sex steroid hormones, allergen exposure, exercise, and other factors that are difficult to control for in vivo murine models (Ursin & Klein, 2021; Williams, 2014; Ying et al., 2022). In this study, male and female slices differed physiologically in the number and size of surfactant C granules and the area of CGRP‐ir fibers. Sex‐related levels of responses to CGRP treatment that included a much larger population of CD19^+^ B cells and cells in NEBs in slices from females compared to males were observed. These neuroimmune sex‐differentiated observations may point to the role of neural signaling in the sex‐dependent outcomes of respiratory disease.

There were some methodological differences between the slice model used here and other PCLS models. Tissue was processed immediately after death instead of 30 min–24 h later. The tissue was never frozen, and no antibacterials/antifungals were used, allowing the lung microbiome to survive. Agarose for inflating the lungs was kept to 2%, which matches some reports but not others. Overall, the time until cutting media was shorter because some steps like tying the trachea closed were omitted. A collagen overlay was used to help keep the slices attached to the dishes. Thickness was held to 250 μm to optimize the nutrient tissue ratio, as per previous experience in other slice paradigms (Tobet et al., 2003). Eight hundred microliters of media were used to help create a clear air/liquid interface at the top of the slices.

In summary, the addition of lung injury levels of CGRP to PCLS results in the proliferation of CD19^+^ B cells, an increase in the size of NEBs, an increase in the number of surfactant C granules, and the disappearance of CGRP immunoreactive fibers. These immunological, physiological, and neurological changes occurred in a sex‐dependent manner. The technical advance of the current slice model allowed the demonstration of neuronal components in the lung that had not been previously demonstrated for CGRP fibers. Using PCLS for neuroimmune lung axis studies allows for exploration of internal lung circuits and will lead to a higher understanding of neuroimmune‐involved disease responses in the future.

## AUTHOR CONTRIBUTIONS

S. Tobet and B. Patlin conceived and designed research; B. Patlin performed experiments, analyzed data, interpreted results of experiments, prepared figures, and drafted the manuscript; L. Schwerdtfeger, B. Patlin, and S. Tobet contributed to starting the project; B. Patlin, L. Schwerdtfeger, and S. Tobet edited and revised the manuscript. S. Tobet funded the project. B. Patlin, L. Schwerdtfeger, and S. Tobet approved the final version of the manuscript.

## FUNDING INFORMATION

S. Tobet and B. Patlin were funded by the Anschutz Pandemic Preparedness Grant, and S. Tobet was partially funded by a U54‐MH118919.

The graphical abstract was partially created with Biorender.com.

## CONFLICT OF INTEREST STATEMENT

No conflicts of interest, financial or otherwise, are declared by the authors.

## ETHICS STATEMENT

All experiments were done in accordance with the Colorado State University Institutional Animal Care and Use Committee (Approval No. 1934).

## Supporting information


**Figure S1:** Tissue slices demonstrated the ability to incorporate exogenous carbohydrate (GalNaz) into mucopolysaccharides, suggesting mucus was actively produced ex vivo. Scale bar is 50 μm.
**Figure S2:** Morphology of lung slices from C57BL6/J Thy1‐YFP transgenic mice across 96 h ex vivo. (a–d) Low magnification images excited at 488 nm are shown compared to higher magnification images (e–h). To separate blood vessels from airways, images taken with the red (550 nm excitation) and the green (488 nm excitation) filter sets on the TE2000‐U inverted microscope were overlayed. This leads to a visualization of blood vessels labeled with fluorescein isothiocyanate in green and airways in yellow due to autofluorescence in the red and green channels. Scale bars are 200 μm.
**Figure S3:** Immunohistochemical labeling of neuronal markers in the lung demonstrates the health of neuronal fibers ex vivo. (a) Peripherin is an intermediate filament protein found in peripheral neurons that serves as a comprehensive marker for neuronal fibers in the lung. The scale bar is 50 μm. (b) immunoreactive choline acetyltransferase (ChAT)‐ir fibers are a marker for cholinergic fibers likely of vagal origin. The scale bar is 25 μm. (c) Substance P‐ir fibers. The scale bar is 100 μm. (d) CGRP‐ir fibers and ir‐CGRP were also found in pulmonary neuroendocrine cells. The scale bar is 25 μm. (e) Vasoactive intestinal peptide (VIP). The white arrows point to fibers that are shown at higher magnification in the inset boxes. Scale bars are 25 μm.
**Figure S4:** There were no significant differences in the average number of B cells in a slice or the average size of a B cell in either sex of slices over 5 days ex vivo. (a–c) Lung slices from males containing B cells from 24‐120 h ex vivo. (d–f) Lung slices from females showing B cells from 24‐120 h ex vivo. (g) There were no significant changes in size or number of B cells in slices from males or females across 24–120 h ex vivo. For male mice, Day 1 *n* = 2 mice/3 PCLS, Day 3 *n* = 2 mice/4 PCLS, and Day 5 *n* = 3 mice/3 PCLS; for female mice, Day 1 *n* = 2 mice/2 PCLS, Day 3 *n* = 3 mice/5 PCLS, and Day 5 *n* = 3 mice/3 PCLS. Scale bars are 50 μm.
**Figure S5:** There were more CGRP‐ir fibers per slice from males than females. (a–c) Slices from male lungs labeled for CGRP showed CGRP‐ir fibers (yellow arrow) and pulmonary neuroendocrine cells (white arrow). (d–f) Slices from female lungs showed B cells after 24, 72, or 120 h ex vivo. (g) There were no significant differences in the average area of CGRP‐ir fibers across time points ex vivo. (h) Slices from male lungs had significantly more CGRP‐ir fibers than females at all time points. White arrows indicate the location of the insets at higher magnification. For male mice, Day 1, Day 3, and Day 5 *n* = 3 mice/4 PCLS; for female mice, Day 1 *n* = 3 mice/3 PCLS, Day 3 *n* = 4 mice/4 PCLS, and Day 5 *n* = 4 mice/4 PCLS. Scale bars are 50 μm.
**Figure S6:** The average area of immunoreactive (ir) peripherin fibers did not differ after 24, 72, or 120 h in slices from males or females. (a–c) Lung slices from males demonstrated ir‐peripherin fibers. (d–f) Lung slices from females demonstrated ir‐peripherin fibers. There were no differences in average fiber area regardless of sex across 24–120 h ex vivo. White arrows indicate ir‐peripherin fibers and the location of the insets at higher magnification. For male mice, Day 1, Day 3, and Day 5 *n* = 3 mice/3 PCLS; for female mice, Day 1 *n* = 3 mice/3 PCLS, Day 3 *n* = 4 mice/4 PCLS, and Day 5 *n* = 4 mice/4 PCLS. Scale bars are 50 μm.
**Figure S7:** Area of ir‐SPC granules was significantly smaller or trended towards being smaller in females than males over 5 days ex vivo. The number and area of granules containing immunoreactive (ir) surfactant C (ir‐SPC) in alveolar type 2 cells were maintained over 5 days ex vivo. (a–c) Slices from male lungs across 24–120 h ex vivo showed ir‐SPC. (d–f) Lung slices from females across 24–120 h ex vivo showing ir‐SPC. (g) ir‐SPC granules in slices from females were significantly smaller at 24 h and 120 h and trended towards being smaller at 72 h. H) No difference in quantities of ir‐SPC granules was observed regardless of sex or time point. For male mice, Day 1, Day 3, and Day 5 *n* = 3 mice/6 PCLS; for female mice, Day 1 *n* = 3 mice/4 PCLS, Day 3 *n* = 3 mice/6 PCLS, and Day 5 *n* = 3 mice/6 PCLS. Scale bars are 50 μm.
**Figure S8:** Tissue slices demonstrated the ability to incorporate exogenous 5‐ethynyl‐2‐deoxyuridine, suggesting that normal proliferation was occurring ex vivo. Bronchiole labeled (B) Scale bar is 100 μm.
