# Supplementary material for: Neuropeptide stimulation of physiological and immunological responses in precision‐cut lung slices
Source: Physiol Rep. 2023 Nov 23;11(22):e15873. doi: 10.14814/phy2.15873 (PMC10665790; doi:10.14814/phy2.15873)

**Supplemental Text**

There were no significant changes in the number of CD19^+^ B cells counted at 24-, 72-, and 120- h ex vivo (P = 0.55). The number of B-cells in slices from females (51 +/- 0.21) was not notably different compared to the number in slices from males (54+/- 0.35). in controls at 24-, 72-, and 120- h ex vivo (Figure S4g; P < 0.22). Representative images of B cell clusters at 24- (Figure S4a, d) 72- (Figure S4b, e), and 120- h (Figure S4c, f) in male (Figure S4d-f) and female (Figure S4a-c) are shown. When compared in size, the size of male (Figure S4d-f) and female (Figure S4a-c) B cells area did not significantly change when measured at 24-, 72-, and 120- h ex vivo P =.97, P=0.36,).

There was no change in area of CGRP fibers measured at 24-, 72-, and 120-h *ex vivo* (P=0.86) and female slices did not exhibit a significant difference in CGRP area per ROI compared to male slices (Figure S3h; P < 0.9; F (4, 27)). No significant difference between male and female CGRP fiber area per ROI (Figure S3g) as shown in male (Figure S5a-c) and female (Figure S5d-f). Compared to total area of CGRP fibers in a male slice, area of female fibers is significantly lower at 24- (P<0.0001) 72-h (P=0.012) and 120-h (Figure S5h; P=0.0045). There was no change in total area per slice (P=0.76) or area per ROI (P=0.82) of Peripherin-ir fibers measured at 24-, 72-, and 120-h *ex vivo*. Female slices did not exhibit a difference (Figure S6g) in fiber area (Figure S6d-f) compared to male slices figure (Figure S6a-c; P=0.26; F (1, 28)).

There was no significant change in size (Figure S7h; P = 0.67) or number (Figure S5g; P = 0.84) of SPC granules seen across five days *ex vivo* seen in males (Figure S7a-c) or females (Figure S7d-f). Male granules were significantly larger than female granules at 24 h (Figure S7a/d; P = 0.011) and 120-h (Figure S7c/f; P = 0.007 and trended towards being larger at 72-h *ex vivo* (Figure S7b/e; P = 0.088).

**Supplemental Figure Cover Page:**

Supplemental Figure 1: Incorporation of sugars to form mucopolysaccharides in live PCLS.

Supplemental Figure 2: Morphology of PCLS

Supplemental Figure 3: neuronal fibers present in PCLS

Supplemental Figure 4: B cells in PCLS taken from male or female mice over 5 days of culture

Supplemental Figure 5: ir-CGRP in PCLS taken from male or female mice over 5 days of culture

Supplemental Figure 6: ir-peripherin in PCLS taken from male or female mice over 5 days of culture

Supplemental Figure 7: ir-SPC in PCLS taken from male or female mice over 5 days of culture

**Supplemental Figures:**

Supplemental Figure 1:


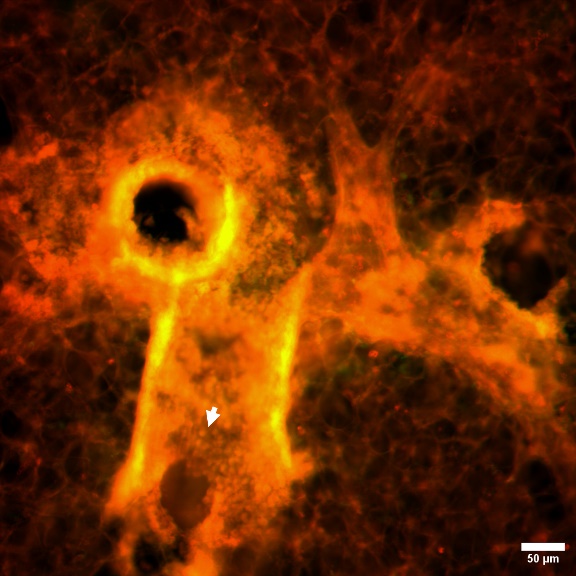


Supplemental Figure 2:
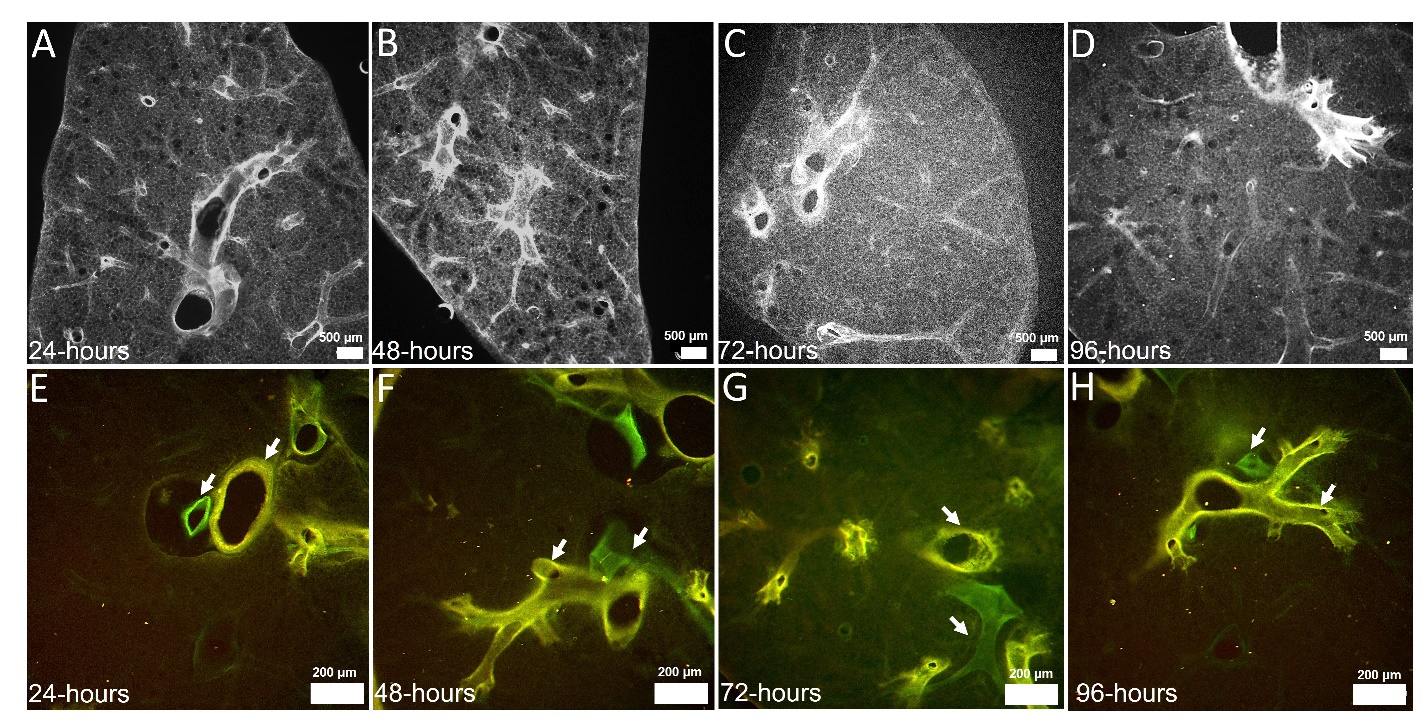


Supplemental Figure 3:
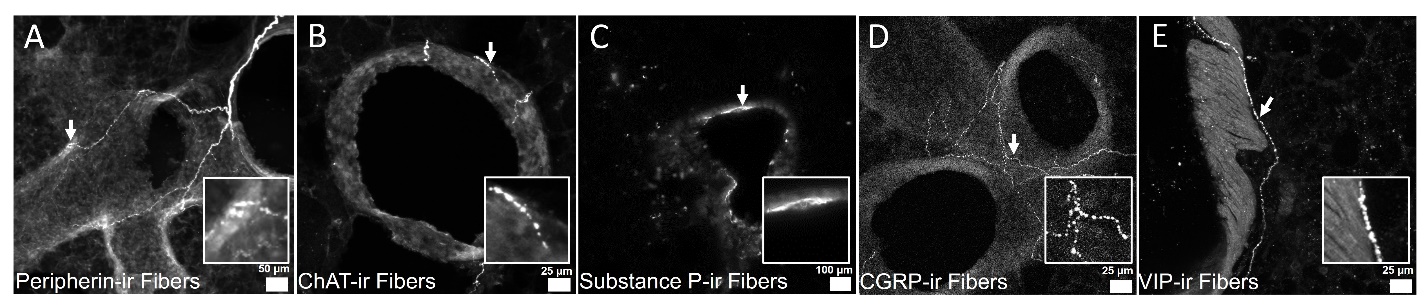


Supplemental Figure 4:
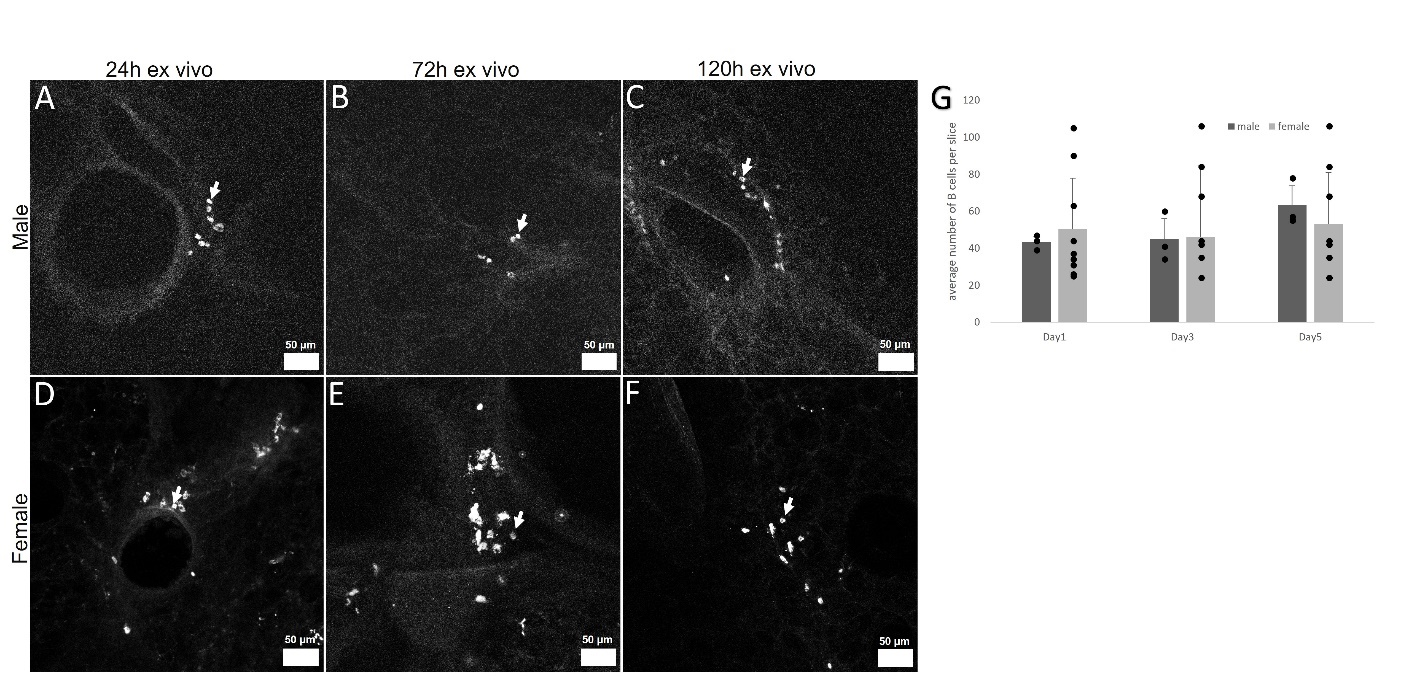


Supplemental Figure 5:
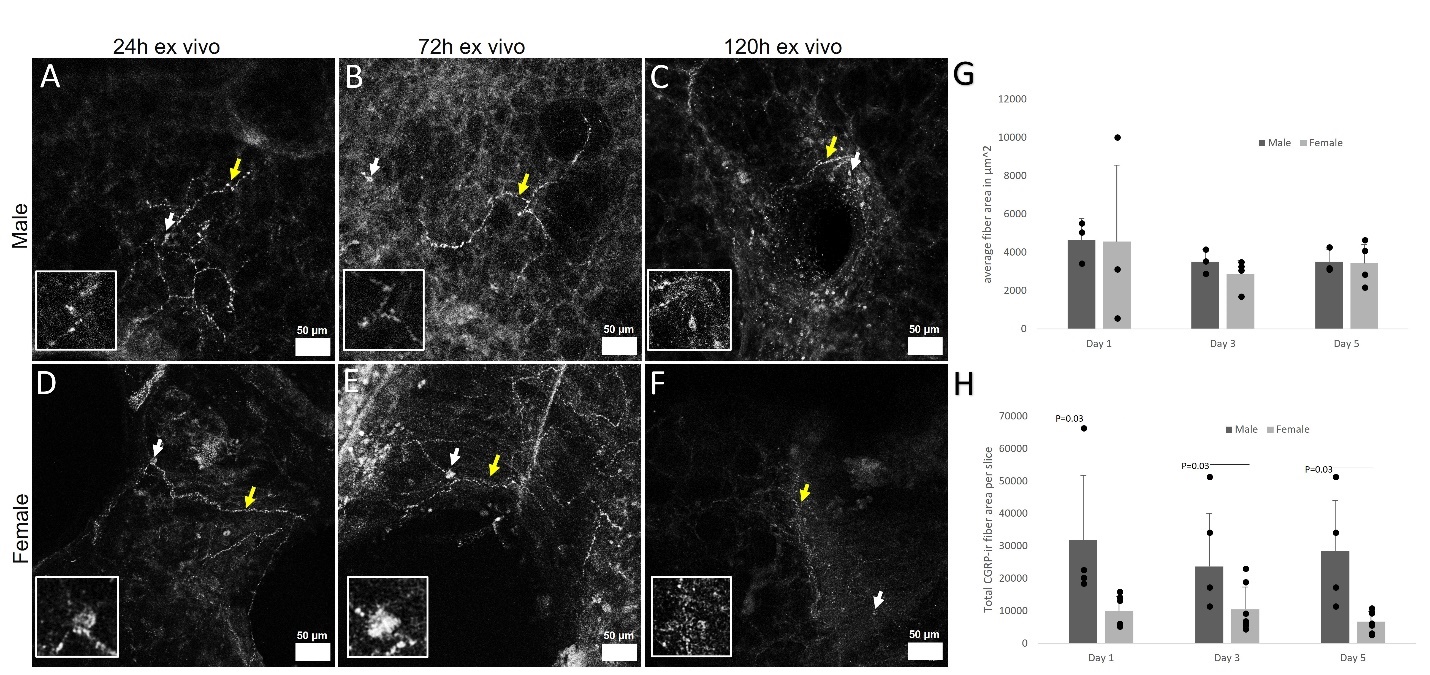


Supplemental Figure 6:
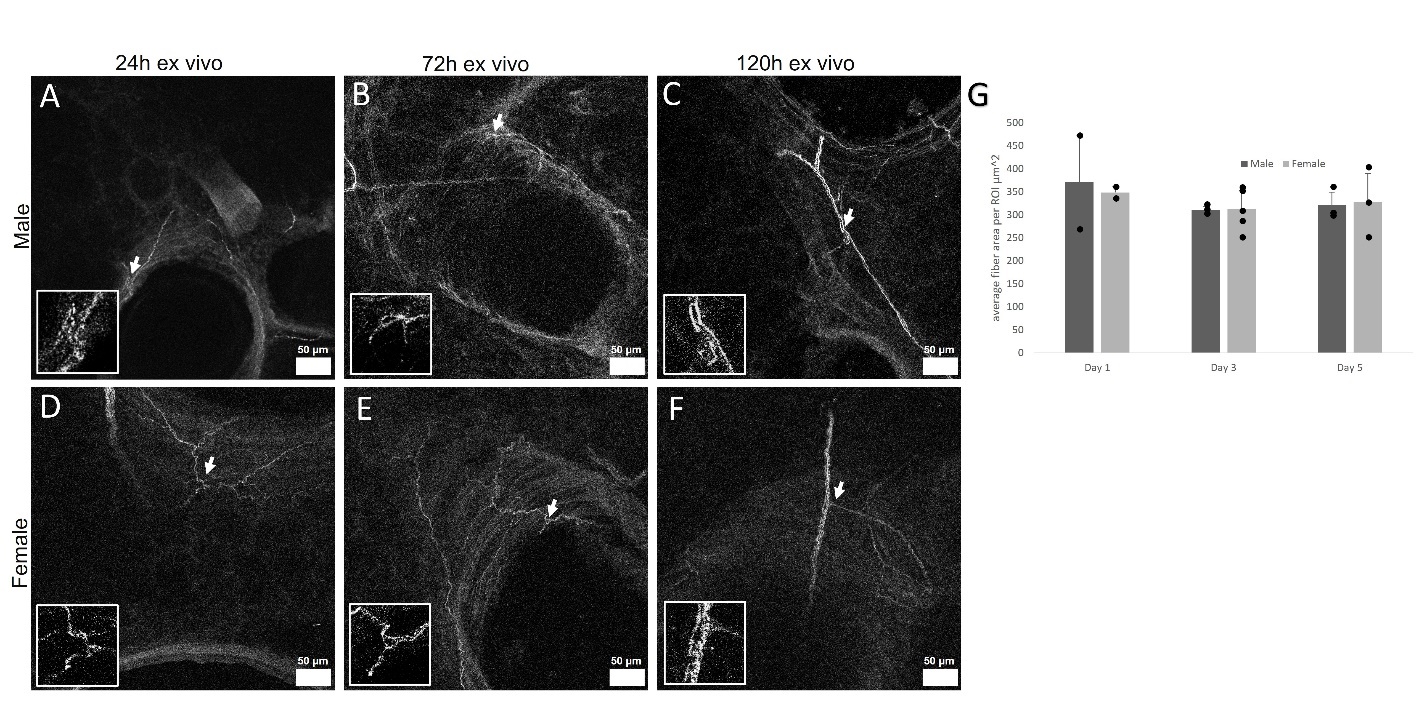


Supplemental Figure 7:
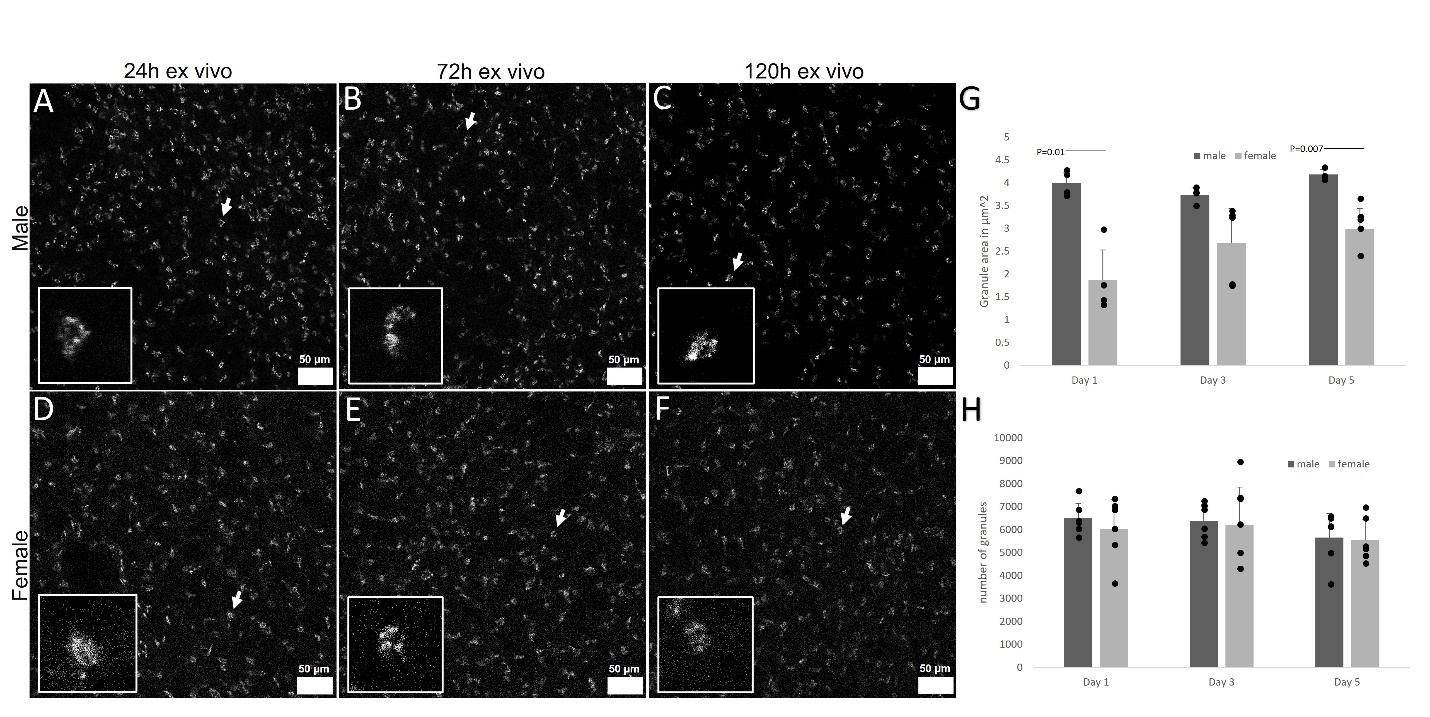


Supplemental Figure 8:
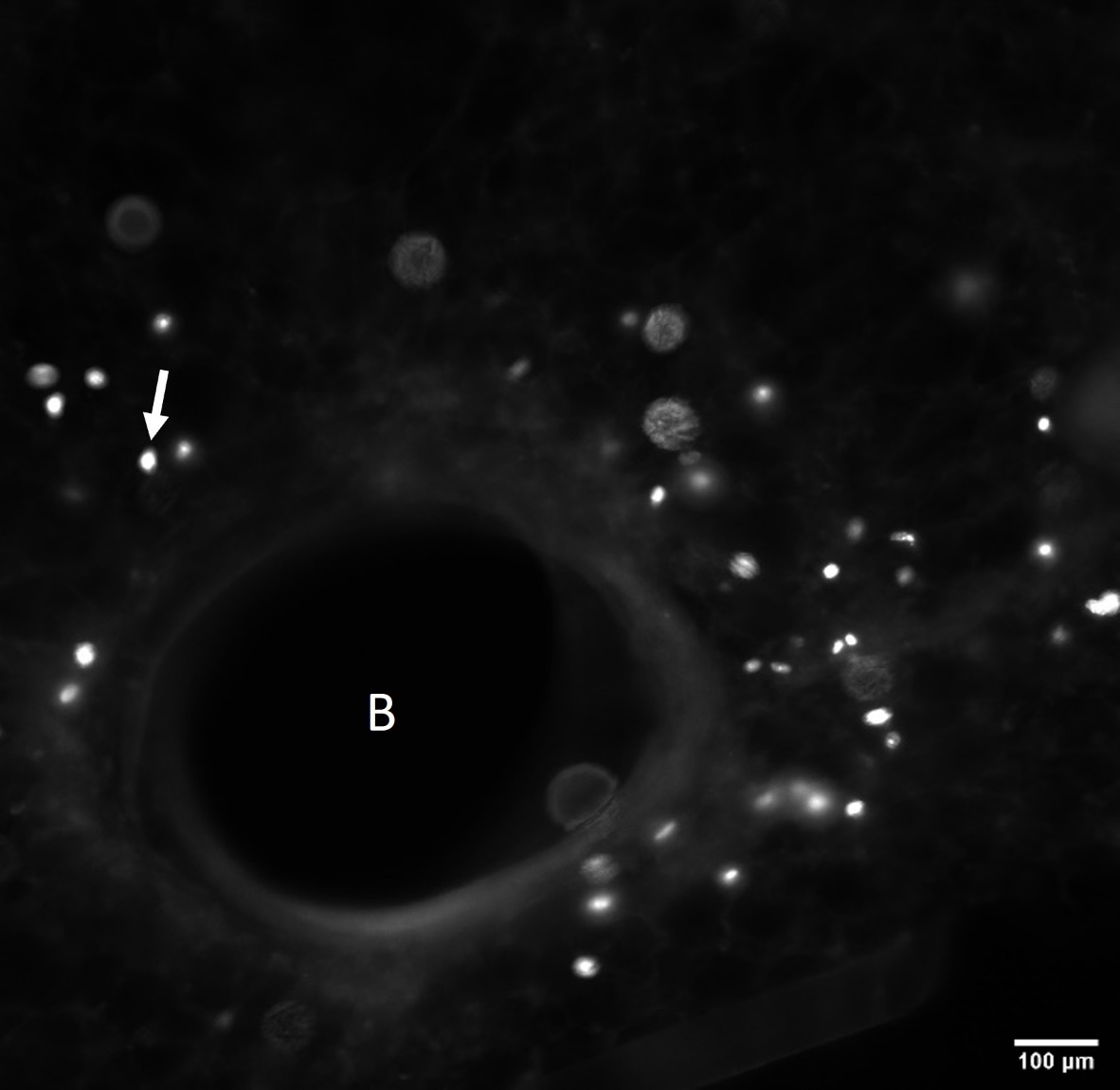

Supplement: Supplementary file 1 — Figure S1: Tissue slices demonstrated the ability to incorporate exogenous carbohydrate (GalNaz) into mucopolysaccharides, suggesting mucus was actively produced ex vivo. Scale bar is 50 μm. Figure S2: Morphology of lung slices from C57BL6/J Thy1‐YFP transgenic mice across 96 h ex vivo. (a–d) Low magnification images excited at 488 nm are shown compared to higher magnification images (e–h). To separate blood vessels from airways, images taken with the red (550 nm excitation) and the green (488 nm excitation) filter sets on the TE2000‐U inverted microscope were overlayed. This leads to a visualization of blood vessels labeled with fluorescein isothiocyanate in green and airways in yellow due to autofluorescence in the red and green channels. Scale bars are 200 μm. Figure S3: Immunohistochemical labeling of neuronal markers in the lung demonstrates the health of neuronal fibers ex vivo. (a) Peripherin is an intermediate filament protein found in peripheral neurons that serves as a comprehensive marker for neuronal fibers in the lung. The scale bar is 50 μm. (b) immunoreactive choline acetyltransferase (ChAT)‐ir fibers are a marker for cholinergic fibers likely of vagal origin. The scale bar is 25 μm. (c) Substance P‐ir fibers. The scale bar is 100 μm. (d) CGRP‐ir fibers and ir‐CGRP were also found in pulmonary neuroendocrine cells. The scale bar is 25 μm. (e) Vasoactive intestinal peptide (VIP). The white arrows point to fibers that are shown at higher magnification in the inset boxes. Scale bars are 25 μm. Figure S4: There were no significant differences in the average number of B cells in a slice or the average size of a B cell in either sex of slices over 5 days ex vivo. (a–c) Lung slices from males containing B cells from 24‐120 h ex vivo. (d–f) Lung slices from females showing B cells from 24‐120 h ex vivo. (g) There were no significant changes in size or number of B cells in slices from males or females across 24–120 h ex vivo. For male mice, Day 1 n = 2 mice/3 [file PHY2-11-e15873-s001.docx]
